# High rates of donor site healing using quadriceps tendon for anterior cruciate ligament reconstruction: A case series

**DOI:** 10.1002/jeo2.12033

**Published:** 2024-06-17

**Authors:** Jérémy Cognault, Pierre‐Fleury Chaillot, Jack Norgate, Jérôme Murgier, International QT Interest Group, Régis Pailhe, Clément Horteur, ReSurg, Chinyelum Agu, Floris van Rooij, Mo Saffarini, Antoine Ponsot

**Affiliations:** ^1^ Clinique du Parc, ELSAN Lyon France; ^2^ Hospices Civils de Lyon Lyon France; ^3^ Clinique Aguilera RAMSAY Santé Biarritz France; ^4^ Clinique Aguilera RAMSAY Santé Biarritz France; ^5^ ReSurg SA Nyon Switzerland

**Keywords:** ACLR, donor site morbidity, QT, ultrasound

## Abstract

**Purpose:**

To investigate the healing of the quadriceps tendon donor site after partial thickness graft harvesting through ultrasound imaging at a short‐term follow‐up of 6‐month following anterior cruciate ligament reconstruction (ACLR) and to investigate the clinical outcomes.

**Methods:**

Between March 2019 and August 2020, 61 knees were retrospectively included in this study. Intraoperatively, the length, width and thickness of the harvested QT graft were measured. At a 6‐month follow‐up, patients were assessed by one of five radiologists, following the same protocol to calculate the defect volume, and patients performed a self‐evaluation of pain on the Visual Analogue Scale, International Knee Documentation Committee (IKDC) and the Knee injury and Osteoarthritis Outcome Scores (KOOS).

**Results:**

Intraoperatively, the QT grafts had a volume of 4635.4 ± 912.5 mm^3^. Postoperatively, ultrasound was performed at 6.5 ± 0.7 months, and the defect volume was 323.3 ± 389.2 mm^3^, representing a healing rate of 93% ± 9% of the donor site. At a minimum 6‐month follow‐up, IKDC was 61.6 ± 16 and KOOS was 70.2 ± 16.6. Age was significantly associated with the healing rate (*β*: −0.005; *p* = 0.032).

**Conclusion:**

At 6 months follow‐up, the defect size of the QT donor site had healed by 93 ± 9% leaving a mean defect volume of 323.3 mm^3^ according to ultrasound measurements. This suggests that the QT has a high capacity for healing after graft harvesting, with 10 patients reaching full defect closure 6 months after surgery. The clinical relevance of these findings is that the quadriceps tendon donor site has high rates of healing, but surgeons should be aware of lower healing rates in older patients.

**Level of Evidence:**

Level IV, retrospective case series.

**Abbreviations**
ACLR: anterior cruciate ligament reconstruction
BMI: body mass index
CSA: cross‐sectional area
HT: hamstring tendon
IKDC: International Knee Documentation Committee
KOOS: Knee injury and Osteoarthritis Outcome Scores
MRI: magnetic resonance imaging
PT: patellar tendon
QT: quadriceps tendon
VAS: Visual Analogue Scale

## INTRODUCTION

Surgical techniques for anterior cruciate ligament reconstruction (ACLR) have developed over the years, resulting in a variety of surgical techniques, and choice of autografts for ACLR, of which the patellar tendon (PT) and hamstring tendon (HT) are the most commonly used [5, 7, 18, 23, 25]. An alternative type of autograft using the quadriceps tendon (QT) was not commonly used during primary ACLR due to the large incision usually required for graft harvesting [2, 19]. Recent literature, however, has shown a growing interest for the use of the QT in ACLR, as it can be harvested using minimally invasive techniques [4], significantly reducing scar size. Furthermore, some studies found that QT may offer greater biomechanical properties, decreased hypaesthesia, pain and irritation compared to PT and HT autografts [1, 4, 11, 17, 18]. In addition, the use of QT resulted in better clinical and functional outcomes in terms of kneeling and squatting [1, 6, 11, 19, 21]. Numerous studies have evaluated the healing of the graft and defect volume of the PT and HT at the harvesting site [13, 14, 15, 24], but to the author's knowledge, no studies have investigated the healing of the QT donor site using ultrasound imaging.

Therefore, the purpose of this study was to investigate the healing of the QT donor site after partial thickness graft harvesting through ultrasound imaging at a short‐term follow‐up of 6 months following surgery. Obtaining measurements of the defect following surgery could provide insight on the healing process. The secondary purpose is to investigate the clinical outcomes after ACLR with QT autograft and the associated factors.

## METHODS

### Patient characteristics

The authors retrospectively studied a consecutive series of patients that underwent ACLR by one senior surgeon between March 2019 and August 2020. Patients were included in the study if they underwent ACLR with a QT autograft and had a minimum follow‐up of 5 months. The criteria for performing ACLR using QT autograft were the availability of healthy intact QT, with no antecedents of trauma, tendinopathy, or surgery. As the study aimed to investigate healing of the QT donor site, rather than the outcomes of ACLR, there were no exclusion criteria, and, therefore, patients with previous ACLR procedures or multiligament injuries were included. All patients provided informed consent prior to surgery for the use of their data for research and publication, the study was approved by the ethical board in advance (IRB approval number: 2022‐11‐COGNAULT‐01) and was performed in accordance with the standards of the 1964 Declaration of Helsinki. The study was conducted following the STROBE guidelines (Supporting Information).

### Preoperative assessment

Preoperative assessment comprised solely of clinical questionnaires. All patients performed a preoperative self‐evaluation assessment using the pain on Visual Analogue Scale (VAS) [3], International Knee Documentation Committee (IKDC) [12] and the Knee injury and Osteoarthritis Outcome Scores (KOOS) with five subcomponents (symptoms, pain, daily activities, sport and quality of life).

### Graft harvesting

**Figure 1:**
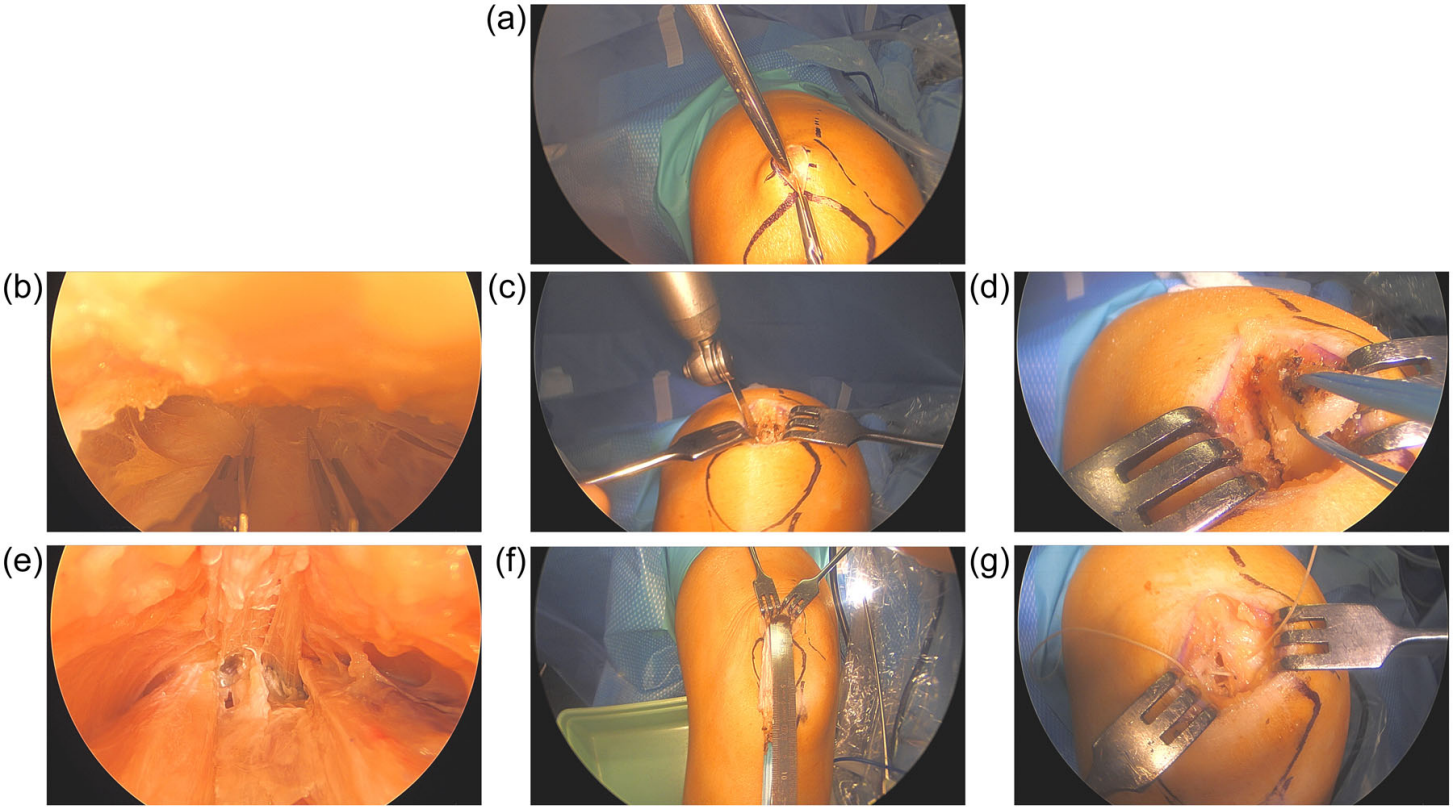
Harvesting of the quadriceps tendon graft.

The surgeon manually assessed the ipsilateral and contralateral knee laxities upon arrival in the operating room. The knee was then positioned at a 90° angle and held in place with an adjustable mechanism attached to the operating table. A tourniquet was placed as proximal as possible on the thigh, with a pressure of 240 mmHg. A 30 mm vertical skin incision was made on the proximal border of the patella (Figure 1a), and using a finger, the QT was detached from the fascia. A 9–10 mm wide double‐edged scalpel was used to mark a QT section of approximately 70–80 mm long (Figure 1b). The length and width of the graft was dependent on the height of the patient and whether it was a primary or revision ACLR, to ensure adequate fixation relative to the potential widening of the pre‐existing tunnel; the graft size was 70 mm long and 9 mm wide for patients <1.90 m and primary ACLR, while it was 80 mm long and 10 mm wide for patients ≥1.90 m or revision ACLR. The exposed patellar bone was then marked with the scalpel to prepare harvesting of a 20 mm long bone block with the same width as the QT section (Figure 1c). A hole was drilled in the middle of the superior part of the bone block. The bone block was separated from the proximal border of the patella using a sagittal saw to cut through both sides of the markings and the distal edge of the block between the two markings. A string was then inserted into the hole to position the block during ACLR (Figure 1d). The surgeon then pulled the bone block and used a sterile ruler to measure a 70–80 mm long QT section, in addition to the 20 mm long bone block (Figure 1e,f), making sure to leave out the tendon of the vastus intermedius, before making the final proximal cut and harvesting the graft. Only partial QT was harvested to facilitate healing and to prevent joint exposure. The quadriceps was then closed using an absorbable suture (Ethicon vicryl plus 5) (Figure 1g).

### Radiologic donor site assessment

**Figure 2:**
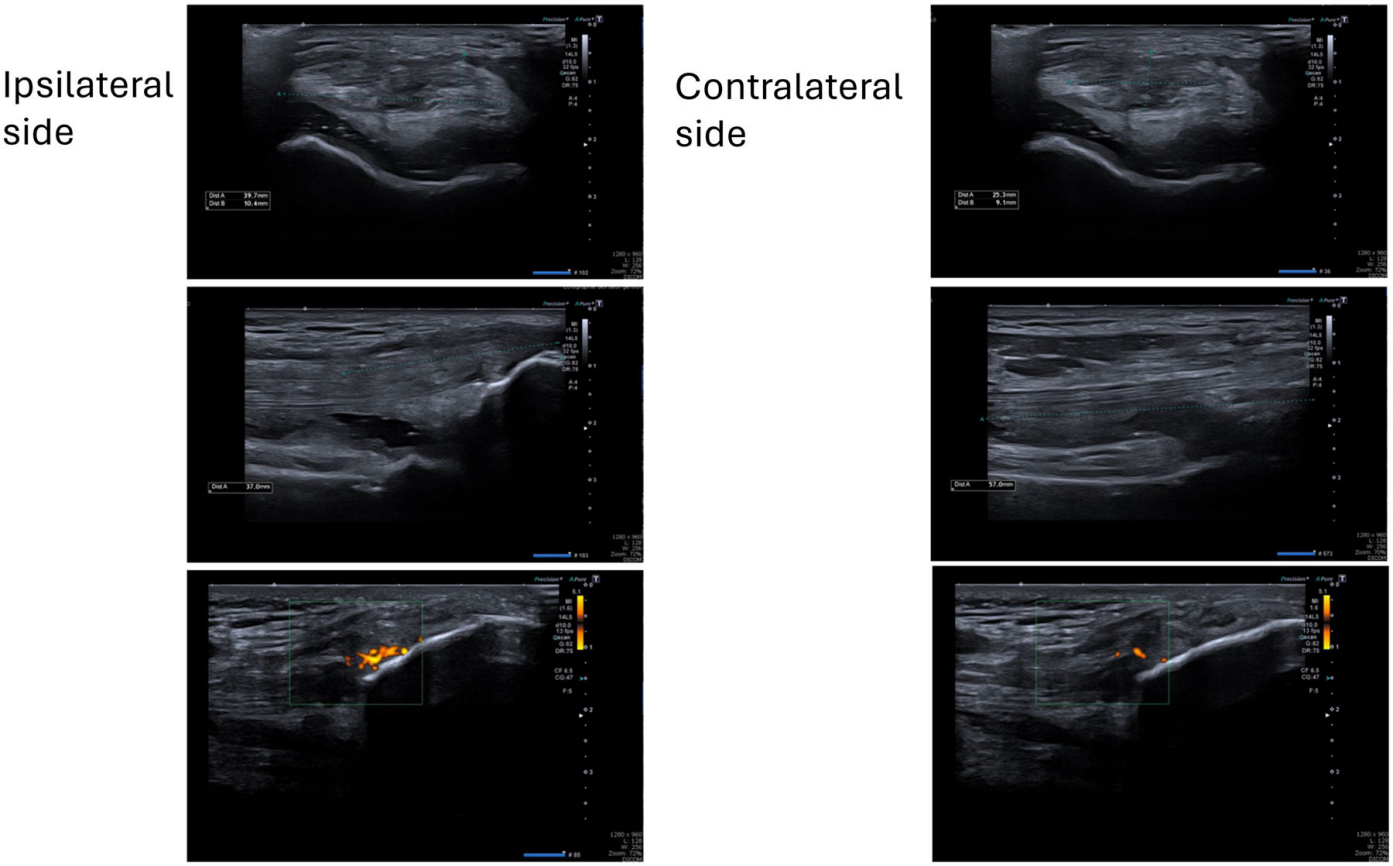
Ultrasound evaluation of the ipsi‐ and contra‐lateral knee.

Standard procedure required patients to be called in at 6 months for a postoperative assessment with the surgeon. Additionally, ultrasound imagery was performed, by five radiologists from the same clinic, at the surgeon's request to observe the healing of the donor site. Patients were assessed by one of the five radiologists, following the same protocol to ensure consistent and reproducible measurements. Additionally, one radiologist verified the measurements of another radiologist for the first two patients allocated to them. The radiologists filled out a standardised form on which they noted the length, width and thickness of the donor site defect to calculate the defect volume. Furthermore, ultrasound was performed on the remaining QT, surrounding the donor site, of the ipsilateral leg (Figure 2) and on the QT of the contralateral leg (Figure 2). The healing of the donor site was evaluated by calculating the decrease in postoperative donor site defect compared to intraoperative graft volume. Other radiologic parameters such as the presence of hyperaemia, failure of healing characterised by liquid components, calcifications and enthesophytes were also noted.

Postoperative assessment at 6 months was deemed necessary to reassure patients with regard to the healing of the QT donor site. As the use of HT or PT is more common, physiotherapists had less experience with QT graft for ACLR, which led to uncertainty concerning the rehabilitation protocol. Performing an ultrasound assessment and communicating the results to the patient encouraged the patients to perform physical exercises to recover from ACLR without worrying about injuring themselves.

### Postoperative clinical assessment

At a minimum follow‐up of 6 months, patients performed a self‐evaluation assessment for pain on VAS, IKDC and KOOS.

### Statistical analysis

Descriptive statistics were used to summarise the findings, and Shapiro–Wilk tests were used to assess the normality of data distributions. For normally distributed continuous data, differences between the ipsi‐ and contralateral leg were evaluated using unpaired *t* tests. Univariable linear regression analyses were performed for postoperative clinical scores, including KOOS, pain on VAS and IKDC, as well as the healing rate, using age, gender, body mass index (BMI), smoking and surgical antecedents as variables. Models were deemed sufficiently powered, considering the recommendations of Austin and Steyerberg of two subjects per variable. Post‐hoc power calculations were also performed, and effect size and the sample size were deemed powerful with a 5% error. Statistical analyses were performed using R version 4.2.3 (R Foundation for Statistical Computing).

## RESULTS

### Patient characteristics

During the study period, 61 knees (61 patients; 37 males and 24 females) met the inclusion criteria. At index surgery, the patients had a mean age of 29.8 ± 10 years (15–54) and a BMI of 24.3 ± 4.3 (18.6–38.8). Of the 61 knees, surgery was performed on 28 right knees (46%) and 33 left knees (54%). Meniscus repairs were required in 33 knees (54%), and four knees underwent revision ACLR (7%). Of the 61 patients, 21 were smokers (34%) (Table 1).

**Table 1:** Patient characteristics.

	*n* = 61	
	Mean ± SD	Range
Female	24 (39%)	
Age	29.4 ± 10	15–54
BMI	24.3 ± 4.3	18.6–39
Smoking	21 (34%)	
Right knee	28 (46%)	
Antecedents		
Meniscus repair	33 (54%)	
Previous injury	13 (21%)	
Revision	4 (7%)	

Abbreviations: BMI, body mass index; SD, standard deviation.

### QT measurements

Intraoperatively, the QT grafts had a mean length of 74.9 ± 6 mm, diameter of 8.8 ± 0.7 mm, cross‐sectional area (CSA) of 61.7 ± 9.8 mm^2^ and volume of 4635.4 ± 912.5 mm^3^. Postoperatively, ultrasound was performed at 6.5 ± 0.7 months (5.0–8.2), and the defect had a mean length of 20.4 ± 16.4 mm, width of 5.6 ± 4.6 mm, thickness of 1.6 ± 0.8 mm and volume of 323.3 ± 389.2 mm^3^, representing a healing rate of 93% ± 9% of the donor site (Table 2), with 10 patients reaching full defect closure 6 months after surgery. The remaining portion of QT surrounding the harvest site on the ipsilateral knee had a mean length of 45.9 ± 12.3 mm, width of 26.9 ± 6.6 mm, thickness of 8.4 ± 2.3 mm and volume of 11,006.7 ± 6372.2 mm^3^. The contralateral QT had a mean length of 44.7 ± 12.0 mm, width of 27.0 ± 6.9 mm, thickness of 7.0 ± 3.2 mm and volume of 8317.1 ± 7125.8 mm^3^ (Table 3).

**Table 2:** Graft information.

	*n* = 61	
	Mean ± SD	Range
Intraoperative		
Graft length (mm)	74.9 ± 6.0	50.0–85.0
Graft cross‐sectional area (mm^2^)	61.7 ± 9.8	38.5–78.5
Graft volume (mm^3^)	4635 ± 912	3079–6675.7

Abbreviation: SD, standard deviation.

**Table 3:** QT measurements on ipsi‐ and contralateral knee 6 months after surgery.

	Ipsilateral knee		Contralateral knee		
	Mean ± SD	Range	Mean ± SD	Range	*p* Value
Time of ultrasound (months)	6.5 ± 0.7	5–8			
Length of the QT (mm)	45.3 ± 13.1	12.0–70.0	44.0 ± 13.0	7.0–70.0	0.67
Width of the QT (mm)	26.9 ± 6.6	15.0–56.0	27.0 ± 6.9	16.0–57.0	0.97
Thickness of the QT (mm)	9.0 ± 5.6	4.0–48.0	7.7 ± 6.3	4.0–47.0	0.23
Volume of the QT (mm)^3^	11,007 ± 6372	0–26400	8317 ± 7126	0–50,460	**0.045**
Defect length (mm)	20.4 ± 16.4	0.0–54.0			
Defect width (mm)	5.6 ± 4.6	0.0–17.0			
Defect thickness (mm)	1.6 ± 0.8	0.0–2.0			
Defect volume (mm^3^)	318 ± 388	0.0–1700.0			
Quadriceps tendon healing	93% ± 9%	52%–100%			

*Note*: Bold value indicates statistically significant differences.

Abbreviations: QT, Quadriceps Tendon; SD, Standard‐Deviation

### Clinical scores

At a minimum follow‐up of 6 months, the pain on VAS score showed a mean net improvement of −0.8 ± 2.4, the IKDC score showed a mean net improvement of 13.2 ± 20 and the mean KOOS score showed a mean net improvement of 15 ± 19.4 (Table 4).

**Table 4:** Clinical scores.

	Preoperative		6 month follow‐up	
	Mean Med ± SD (IQR)	Range	Mean Med ± SD (IQR)	Range
VAS scores	2.2 ± 2.3	0–8.6	1.5 ± 1.4	0–5.9
IKDC scores	48.6 ± 14.2	13.8–90.8	61.6 ± 16	23.0–93.1
KOOS scores				
Pain	70.3 ± 17.6	25–100	80.1 ± 13.3	27.8–100
Symptoms	68.5 ± 15.3	32.1–100	71.1 ± 17	17.9–100
ADL	76.1 ± 18.4	16.2–100	89.2 ± 11	44.1–100
Sports	33.6 ± 26.7	0–95.0	54.4 ± 24.5	0–100
QoL	29.6 ± 19.5	0–7.5	53.5 ± 19.8	6.3–100
Mean Score	56 ± 16.8	21–95.1	71 ± 16.7	26–99.3

Abbreviations: IKDC, International Knee Documentation Committee; IQR, Interquartile range; KOOS, Knee injury and Osteoarthritis Outcome Scores; Med, median; SD, standard deviation; VAS, Visual Analogue Scale (for pain).

### Secondary outcomes

A liquid component was observed in eight patients (13%), while three (5%) other patients developed calcifications and 12 (20%) had enthesophytes. Hyperaemia was detected in 43 (70%) patients using the Doppler effect with the ultrasound.

### Univariable analysis

Age was significantly associated with the healing rate (*β*: −0.25; 95% confidence interval [CI]: −0.48 to 0.02; *p* = 0.032), while BMI was significantly associated to pain on VAS (*β*: 0.11; 95% CI: 0.02 to 0.19; *p* = 0.013) and IKDC (*β*: 1.12; 95% CI: −2.13 to −0.10); *p* = 0.032) (Table 5).

**Table 5:** Univariable regression analysis.

	Healing rate (*n* = 61)			Postoperative KOOS (*n* = 45)			Postoperative VAS pain score (*n* = 45)			Postoperative IKDC subjective score (*n* = 43)		
Variable	*β*	95% CI	*p* Value	*β*	95% CI	*p* Value	*β*	95% CI	*p* Value	*β*	95% CI	*p* Value
Age	−0.25	−0.48 to 0.02	**0.032**	7.34	3.40 to 11.28	**<0.001**	−0.07	−0.11 to −0.03	**<0.001**	0.82	0.38 to 1.26	**<0.001**
Sex	2.57	−2.27 to 7.41	0.292	−50.44	−140.6 to 39.72	0.267	−0.57	−1.42 to 0.28	0.183	1.60	−8.53 to 11.73	0.751
BMI	0.23	−0.32 to 0.79	0.401	7.22	−3.79 to 18.23	0.194	−0.08	−0.17 to 0.01	0.080	0.32	−0.75 to 1.39	0.549
Smoking	3.12	−1.84 to 8.07	0.213	24.81	−69.30 to 118.92	0.599	−0.69	−1.56 to 0.18	0.116	−0.67	−11.16 to 9.83	0.898
Surgical antecedents	0.27	−4.55 to 5.10	0.911	62.47	−25.34 to 150.29	0.159	0.04	−0.85 to 0.93	0.931	−4.75	−15.14 to 5.64	0.362

*Note*: Bold values indicate statistically significant differences.

Abbreviations: BMI, body mass index; CI, confidence interval.

## DISCUSSION

The most important finding for this study is that at a minimum follow‐up of 5 months, the defect size of the donor site had healed by 93 ± 9% leaving a mean defect volume of 323.3 mm^3^ according to ultrasound measurements of the donor site. This suggests that the QT has a high capacity for regeneration after graft harvesting, with 10 patients reaching full defect closure 6 months after surgery. Furthermore, the univariable analysis revealed that age was negatively associated with the healing rate. The clinical relevance of these findings is that the QT donor site has high rates of healing, but surgeons should be aware of lower healing rates in older patients, although further studies are required to determine the impact of this finding. To the author's knowledge, no studies have investigated the healing of the QT donor site using ultrasound imaging, and therefore this study could serve as reassurance for physiotherapists and patients who are receiving ACLR with a QT graft, as the healing process and the risk of tear is less known that other graft types.

Similar studies on the healing process of the PT donor site using ultrasound [26] or magnetic resonance imaging (MRI) [14, 24] have been published. In 2015, Yazdanshenas et al. [26] published a study investigating the donor site healing for the PT at 6 and 12 months following surgery using ultrasound and found that 70% of the patients recovered after 6 months and 100% after 12 months. Healing was assessed by comparing the echogenicity of the contralateral PT to the donor site. In 2000, Kartus et al. [14] compared MRI to ultrasound for PT donor site healing, and while MRI was unable to detect a residual gap in 16% of the patients, ultrasound showed no patients had a fully healed PT. Recent literature has shown that ultrasound is better for superficial soft tissue assessment than MRI [8] and has the added benefit of being easier to perform. In addition, Kartus et al. [14] found that the width and thickness of the ipsilateral donor site was greater than the contralateral side, which is in agreement to the findings of the present study, in which we found that the width of the remaining QT was comparable between the ipsi‐ and contralateral side while the length and thickness were greater for the ipsilateral side. These findings could be explained by the postoperative swelling of the tendon, in addition to newly formed scar tissue at the QT graft harvest site.

Numerous studies have investigated whether there is an association between clinical scores and graft choice, however, little to no differences were found [1, 6, 11, 14, 16, 19, 24]. Mouarbes et al. [19] reported a statistically significant larger area of hypaesthesia for both the PT and HT compared to the QT, and comparable findings were published by Horteur et al. [9]. In a study published by Runer et al. [22], QT and HT clinical scores were assessed at multiple time points over a 6‐year follow‐up. Pain on VAS was comparable for QT and HT at 6 months follow‐up (1.3 ± 1.6 vs. 0.9 ± 0.8) and at 60 months follow‐up (0.5 ± 0.9 vs. 0.6 ± 1.0). Runer et al. [22] also found equivalent IKDC and KOOS for QT and HT at final follow‐up. In the present study, we found a KOOS score of 70.2 ± 16.6, IKDC of 61.6 ± 16 and pain on VAS of 1.5 ± 1.4 at 6 months follow‐up, which could further improve at longer term follow‐up.

The QT shows reliable results and can be considered as an alternative to the PT and HT for ACLR, and QT offers better outcomes in terms of kneeling and squatting [10, 21], which may be due to the healing rate at 6 months after surgery. This implies rapid and efficient regeneration of the tissue and minimal impact to the extensor mechanism. The QT's capacity to withstand loads and effort appears restored with the reduction of the donor site defect.

In addition to the functional qualities of the QT graft, the literature shows high patient satisfaction with this choice of graft with the exception of incision size and scar aesthetics [11]. While QT graft harvesting techniques used to require large incisions to proximally detach the graft from the tendon, leaving a lengthy scar on the anterior thigh, new techniques are available that only require a 3 cm vertical incision made possible by the careful pressure application of the tourniquet [20]. Applying 240 mmHg suppresses the blood flow, yet does not constrict the QT, maintaining enough muscle elasticity to pull the tendon through the incision up to the desired graft length before fully releasing the graft.

The main limitation of this study is the short‐term follow‐up. A longer observation period is required to analyse the full QT healing process and observe the potential residual scarring of the QT once the defect has healed in all patients, along with possible side effects. A longer follow‐up could allow to evaluate the evolution of the clinical scores. Additionally, while width and thickness could be assessed with no difficulty using ultrasound, in the present study, defect length was limited by the size of the ultrasound wand, resulting in shorter defect lengths than expected. Further studies are required with more adequate equipment to continue investigating the healing process of the QT. Furthermore, patients were not in the same position when harvesting the graft and when performing the ultrasound measurement at 6 months and were assessed by five different radiologists post‐operatively. Standardising the measurement methods would remove any potential biases for future studies. Finally, to protect the graft, no functional assessment was performed at 6 postoperative months, and, therefore, longer term follow‐up could include this assessment.

## CONCLUSION

At 6 months follow‐up, the defect size of the QT donor site had healed by 93 ± 9% leaving a mean defect volume of 323.3 mm^3^ according to ultrasound measurements. This suggests that the QT has a high capacity for healing after graft harvesting, with 10 patients reaching full defect closure 6 months after surgery. The clinical relevance of these findings is that the QT donor site has high rates of healing, but surgeons should be aware of lower healing rates in older patients.

## CONTRIBUTORS OF International QT Interest Group

Régis Pailhe (Clinique Aguilera, RAMSAY Santé, Biarritz, France); Clément Horteur (CHU de Grenoble, Grenoble, France).

## CONTRIBUTORS OF ReSurg

Chinyelum Agu, Floris van Rooij and Mo Saffarini (ReSurg SA, Nyon, Switzerland).

## AUTHOR CONTRIBUTIONS

**Jérémy Cognault**: Procurement of funding; study design; data collection; interpretation of findings and manuscript editing. **Pierre‐Fleury Chaillot**: Study design; data collection; interpretation of findings and manuscript validation. **Jack Norgate**: Study design; data collection; interpretation of findings and manuscript validation. **Jérôme Murgier**: Study design; interpretation of findings and manuscript validation. **Régis Pailhe**: Study design; interpretation of findings and manuscript validation. **Clément Horteur**: Study design; interpretation of findings and manuscript validation. **Chinyelum Agu**: Methodology; data curation; formal analysis; statistical analysis; manuscript writing. **Floris van Rooij**: Methodology; data curation; formal analysis; manuscript writing. **Mo Saffarini**: Methodology; supervision; interpretation of findings. **Antoine Ponsot**: Study design; data collection; interpretation of findings and manuscript validation. All authors approved the final manuscript.

## CONFLICT OF INTEREST STATEMENT

The authors declare no conflict of interest.

## ETHICS STATEMENT

This study was performed in line with the principles of the Declaration of Helsinki and was approved in advance by the institutional review board. All patients provided informed consent for the analysis and use of their data, and the study.

## Supporting information

Supporting information.

## Data Availability

Data are available upon reasonable request.

## References

[1] Barié, A. , Sprinckstub, T. , Huber, J. & Jaber, A. (2020) Quadriceps tendon vs. patellar tendon autograft for ACL reconstruction using a hardware‐free press‐fit fixation technique: comparable stability, function and return‐to‐sport level but less donor site morbidity in athletes after 10 years. Archives of Orthopaedic and Trauma Surgery, 140(10), 1465–1474. Available from: 10.1007/s00402-020-03508-1 32504178 PMC7505888

[2] Bartlett, R.J. , Clatworthy, M.G. & Nguyen, T.N.V. (2001) Graft selection in reconstruction of the anterior cruciate ligament. The Journal of Bone and Joint Surgery. British Volume, 83(5), 625–634. Available from: 10.1302/0301-620X.83B5.0830625 11476294

[3] Bullens, P.H.J. , van Loon, C.J.M. , de Waal Malefijt, M.C. , Laan, R.F.J.M. & Veth, R.P.H. (2001) Patient satisfaction after total knee arthroplasty. The Journal of Arthroplasty, 16(6), 740–747. Available from: 10.1054/arth.2001.23922 11547372

[4] Clinger, B. , Xerogeanes, J. , Feller, J. , Fink, C. , Runer, A. , Richter, D. et al. (2022) Quadriceps tendon autograft for anterior cruciate ligament reconstruction: state of the art. Journal of ISAKOS, 7(6), 162–172. Available from: 10.1016/j.jisako.2022.08.010 36096362

[5] D'Ambrosi, R. , Meena, A. , Raj, A. , Ursino, N. , Formica, M. , Herbort, M. et al. (2023) Multiple revision anterior cruciate ligament reconstruction: not the best but still good. Knee Surgery, Sports Traumatology, Arthroscopy, 31(2), 559–571. Available from: 10.1007/s00167-022-07197-8 PMC9898374 36224291

[6] Dai, W. , Leng, X. , Wang, J. , Cheng, J. , Hu, X. & Ao, Y. (2022) Quadriceps tendon autograft versus bone‐patellar tendon‐bone and hamstring tendon autografts for anterior cruciate ligament reconstruction: a systematic review and meta‐analysis. The American Journal of Sports Medicine, 50(12), 3425–3439. Available from: 10.1177/03635465211030259 34494906

[7] Delay, B.S. , Smolinski, R.J. , Wind, W.M. & Bowman, D.S. (2001) Current practices and opinions in ACL reconstruction and rehabilitation: results of a survey of the American Orthopaedic Society for Sports Medicine. The American Journal of Knee Surgery, 14(2), 85–91. 11401175

[8] Fritz, B. & Fritz, J. (2023) MR imaging‐ultrasonography correlation of acute and chronic foot and ankle conditions. Magnetic Resonance Imaging Clinics of North America, 31(2), 321–335. Available from: 10.1016/j.mric.2023.01.009 37019553

[9] Horteur, C. , Cavalié, G. , Gaulin, B. , Cohen Bacry, M. , Morin, V. , Cavaignac, E. et al. (2020) Saphenous nerve injury after anterior cruciate ligament reconstruction: reduced numbness area after ligamentoplasty using quadriceps tendon compared with hamstring tendon. The Knee, 27(4), 1151–1157. Available from: 10.1016/j.knee.2020.05.020 32711876

[10] Horteur, C. , Rubens Duval, B. , Merlin, A. , Cognault, J. , Ollivier, M. & Pailhe, R. (2022) Comparison of knee extensor strength after anterior cruciate ligament reconstruction using either quadriceps tendon or hamstring tendon autografts. European Journal of Orthopaedic Surgery & Traumatology, 32(5), 857–865. Available from: 10.1007/s00590-021-03062-5 34152474

[11] Hurley, E.T. , Mojica, E.S. , Kanakamedala, A.C. , Meislin, R.J. , Strauss, E.J. , Campbell, K.A. et al. (2022) Quadriceps tendon has a lower re‐rupture rate than hamstring tendon autograft for anterior cruciate ligament reconstruction—a meta‐analysis. Journal of ISAKOS, 7(2), 87–93. Available from: 10.1016/j.jisako.2021.10.001 35543668

[12] Irrgang, J.J. , Anderson, A.F. , Boland, A.L. , Harner, C.D. , Kurosaka, M. , Neyret, P. et al. (2001) Development and validation of the international knee documentation committee subjective knee form. The American Journal of Sports Medicine, 29(5), 600–613. Available from: 10.1177/03635465010290051301 11573919

[13] Kanamoto, T. , Tanaka, Y. , Yonetani, Y. , Kita, K. , Amano, H. , Ueda, Y. et al. (2023) Changes in patellar height after anatomical ACL reconstruction with BTB autograft with a focus on patellar tendon removal volume. Journal of Orthopaedic Science, 28(2), 403–407. Available from: 10.1016/j.jos.2021.12.007 34996699

[14] Kartus, J. , Movin, T. , Papadogiannakis, N. , Christensen, L.R. , Lindahl, S. & Karlsson, J. (2000) A radiographic and histologic evaluation of the patellar tendon after harvesting its central third. The American Journal of Sports Medicine, 28(2), 218–226. Available from: 10.1177/03635465000280021301 10750999

[15] Kiss, Z.S. , Kellaway, D.P. , Cook, J.L. & Khan, K.M. (1998) Postoperative patellar tendon healing: an ultrasound study. Australasian Radiology, 42(1), 28–32. Available from: 10.1111/j.1440-1673.1998.tb00559.x 9509600

[16] Lee, D.W. , Lee, J. , Jang, S. , Ro, D.H. , Lee, M.C. & Han, H.S. (2021) Long‐term outcomes of anterior cruciate ligament reconstruction using quadriceps tendon‐patellar bone autograft. Orthopaedic Journal of Sports Medicine, 9(6), 232596712110174. Available from: 10.1177/23259671211017474 PMC8193668 34179211

[17] Malinowski, K. , Paszkowski, J. , Mostowy, M. , Góralczyk, A. , LaPrade, R.F. & Hermanowicz, K. (2021) Quadriceps tendon‐bone full‐thickness autograft: reproducible and easy harvesting technique using simple surgical tools. Arthroscopy Techniques, 10(4), e1165–e1172. Available from: 10.1016/j.eats.2021.01.003 33981566 PMC8085438

[18] Meena, A. , D'Ambrosi, R. , Runer, A. , Raj, A. , Attri, M. , Abermann, E. et al. (2023) Quadriceps tendon autograft with or without bone block have comparable clinical outcomes, complications and revision rate for ACL reconstruction: a systematic review. Knee Surgery, Sports Traumatology, Arthroscopy, 31(6), 2274–2288. Available from: 10.1007/s00167-022-07281-z PMC10183433 36534150

[19] Mouarbes, D. , Dagneaux, L. , Olivier, M. , Lavoue, V. , Peque, E. , Berard, E. et al. (2020) Lower donor‐site morbidity using QT autografts for ACL reconstruction. Knee Surgery, Sports Traumatology, Arthroscopy, 28(8), 2558–2566. Available from: 10.1007/s00167-020-05873-1 32020251

[20] Ollivier, M. , Cognault, J. , Pailhé, R. , Bayle‐Iniguez, X. , Cavaignac, E. & Murgier, J. (2021) Minimally invasive harvesting of the quadriceps tendon: technical note. Orthopaedics & Traumatology: Surgery & Research, 107(2), 102819. Available from: 10.1016/j.otsr.2021.102819 33497791

[21] Riaz, O. , Aqil, A. , Mannan, A. , Hossain, F. , Ali, M. , Chakrabarty, G. et al. (2018) Quadriceps tendon‐bone or patellar tendon‐bone autografts when reconstructing the anterior cruciate ligament: a meta‐analysis. Clinical Journal of Sport Medicine, 28(3), 316–324. Available from: 10.1097/JSM.0000000000000451 28654440

[22] Runer, A. , Suter, A. , Roberti di Sarsina, T. , Jucho, L. , Gföller, P. , Csapo, R. et al. (2023) Quadriceps tendon autograft for primary anterior cruciate ligament reconstruction show comparable clinical, functional, and patient‐reported outcome measures, but lower donor‐site morbidity compared with hamstring tendon autograft: a matched‐pairs study with a mean follow‐up of 6.5 years. Journal of ISAKOS, 8(2), 60–67. Available from: 10.1016/j.jisako.2022.08.008 36216218

[23] Singh, H. , Glassman, I. , Sheean, A. , Hoshino, Y. , Nagai, K. & de Sa, D. (2023) Less than 1% risk of donor‐site quadriceps tendon rupture post‐ACL reconstruction with quadriceps tendon autograft: a systematic review. Knee Surgery, Sports Traumatology, Arthroscopy, 31(2), 572–585. Available from: 10.1007/s00167-022-07175-0 36255474

[24] Svensson, M. , Kartus, J. , Ejerhed, L. , Lindahl, S. & Karlsson, J. (2004) Does the patellar tendon normalize after harvesting its central third?: a prospective long‐term MRI study. The American Journal of Sports Medicine, 32(1), 34–38. Available from: 10.1177/0363546503258935 14754721

[25] Xerogeanes, J.W. , Mitchell, P.M. , Karasev, P.A. , Kolesov, I.A. & Romine, S.E. (2013) Anatomic and morphological evaluation of the quadriceps tendon using 3‐dimensional magnetic resonance imaging reconstruction: applications for anterior cruciate ligament autograft choice and procurement. The American Journal of Sports Medicine, 41(10), 2392–2399. Available from: 10.1177/0363546513496626 23893419

[26] Yazdanshenas, H. , Madadi, F. , Madadi, F. , Washington 3rd, E.R. , Jones, K. & Shamie, A.N. (2015) Patellar tendon donor‐site healing during six and twelve months after anterior cruciate ligament reconstruction. Journal of Orthopaedics, 12(4), 179–183. Available from: 10.1016/j.jor.2015.05.018 26566316 PMC4602002

