# Supplementary material for: High rates of donor site healing using quadriceps tendon for anterior cruciate ligament reconstruction: A case series
Source: J Exp Orthop. 2024 Jun 17;11(3):e12033. doi: 10.1002/jeo2.12033 (PMC11180971; doi:10.1002/jeo2.12033)
Supplement: Supplementary file 1 — Supporting information. [file JEO2-11-e12033-s001.docx]

STROBE Statement—checklist of items that should be included in reports of observational studies

|  | Item No. | Recommendation | Page  No. | Relevant text from manuscript |
| --- | --- | --- | --- | --- |
| **Title and abstract** | 1 | (*a*) Indicate the study’s design with a commonly used term in the title or the abstract | 1 | High rates of donor site healing using quadriceps tendon for anterior cruciate ligament reconstruction: a case series |
|  |  | (*b*) Provide in the abstract an informative and balanced summary of what was done and what was found | 2 | **Methods**: Between March 2019 and August 2020, 61 knees (37 males and 24 females) were retrospectively included in this study. Intraoperatively, the length, width and thickness of the harvested quadriceps tendonQT graft were measured. At a 6- month follow-up, 5 radiologists from the same clinic performed the ultrasound analysis patients were assessed by 1 of 5 radiologists, following the same protocol and filled out a standardized form reporting the length, width, and thickness of the donor site defect to calculate the defect volume, and . pAt a minimum follow-up of 6 months, patients performed a self-evaluation assessment using theof pain on Visual Analogue Scale (VAS), International Knee Documentation Committee (IKDC), the Knee injury and Osteoarthritis Outcome Scores (KOOS)., the Western Ontario and McMaster Universities Osteoarthritis Index (WOMAC), the Tegner activity scale, the Single Assessment Numeric Evaluation (SANE), the Veterans RAND 12-item Health Survey (VR-12), and the Anterior Cruciate Ligament Return to Sport After Injury (ACL-RSI) Scale.  **Results**: At index surgeryIntraoperatively, the patients had a mean age of 29.8±10 years, and a body mass index (BMI) of 24.3±4.3. Intraoperatively, the quadriceps tendonQT grafts had a mean length of 74.9±6mm, a cross-sectional area of 61.7±9.8mm2, and volume of 4635.4±912.5mm3. Postoperatively, ultrasound was performed at 6.5±0.7 months, and the defect volume was 323.3±389.2mm3, representing a healing rate of 93%±9% of the donor site. At a minimum 6-month follow-up of 6 months, the mean IKDC score was 61.6±16 and the mean KOOS score was 70.2±16.6. Age was significantly associated with the healing rate (β,-0.005; p=0.032).  **Conclusion**: At 6 months follow up, the defect size of the QT donor site had healed by 93±9% leaving a mean defect volume of 323.3mm3 according to ultrasound measurements. This suggests that the QT has a high capacity for healing after graft harvesting, with 10 patients reaching full defect closure 6 months after surgery. The clinical relevance of these findings is that the Qquadriceps tendon donor site has high rates of healing, but surgeons should be aware of lower healing rates in older patients. |
| Introduction | | | |  |
| Background/rationale | 2 | Explain the scientific background and rationale for the investigation being reported | 4 | Surgical techniques for anterior cruciate ligament reconstruction (ACLR) have developed over the years, resulting in a variety of surgical techniques, relative to the operating surgeon’s preferences and methods, but also choice of graft. Nowadays, surgeons have the and choice of using various autografts for ACLR, of which the patellar tendon (PT) and hamstring tendon (HT) are the most commonly used due to the reliability of the harvesting technique and required quality of the graft. An alternative type of autograft was proposed using the quadriceps tendon (QT), but was not commonly used during primary ACLR due to the large incision usually required for graft harvesting .  Recent literature, however, has shown a growing interest for the use of the QT in ACLR, as it can be harvested using minimally invasive techniques, significantly reducing scar size. Furthermore, some studies found that QT may offer greater biomechanical properties, decreased hypoesthesia, pain and irritation compared to PT and HT autografts. In addition to this, the use of QT resulted in better clinical and functional outcomes in terms of kneeling and squatting |
| Objectives | 3 | State specific objectives, including any prespecified hypotheses |  | Therefore, the purpose of this study was to investigate the healing of the QT donor site after partial thickness graft harvesting through ultrasound imaging at a short-term follow-up of 6 months following surgery. Obtaining measurements of the defect following surgery could provide insight on the healing process. The secondary purpose is to investigate the clinical recovery after ACLR with QT autograft and the factors associated with it. |
| Methods | | | |  |
| Study design | 4 | Present key elements of study design early in the paper | 5 | The authors retrospectively studied a consecutive series of 61 patients (61 knees) that received ACLR using QT autograft by one senior surgeon between March 2019 and August 2020. |
| Setting | 5 | Describe the setting, locations, and relevant dates, including periods of recruitment, exposure, follow-up, and data collection | 5 | The authors retrospectively studied a consecutive series of patients that underwent ACLR by one senior surgeon between March 2019 and August 2020. Patients were included in the study if they underwent ACLR with a QT autograft, and had a minimum follow-up of 5 months. The authors studied a consecutive series of 61 patients (61 knees) that received ACLR using QT autograft by one surgeon between March 2019 and August 2020.The criteria for performing ACLR using QT autograft were the availability of healthy intact QT, with no antecedents of trauma, tendinopathy, or surgery. |
| Participants | 6 | (*a*) *Cohort study*—Give the eligibility criteria, and the sources and methods of selection of participants. Describe methods of follow-up  *Case-control study*—Give the eligibility criteria, and the sources and methods of case ascertainment and control selection. Give the rationale for the choice of cases and controls  *Cross-sectional study*—Give the eligibility criteria, and the sources and methods of selection of participants | 5 | As the study aimed to investigate healing of the QT donor site, rather than the outcomes of ACLR, there were no exclusion criteria, and therefore patients with The series included 3 knees that had previous ACLR procedures or and 2 knees with multiligament injuries , which were includednot excluded, as the study aimed to investigate healing of the QT donor site at 6 months, rather than the outcomes of ACLR |
|  |  | (*b*) *Cohort study*—For matched studies, give matching criteria and number of exposed and unexposed  *Case-control study*—For matched studies, give matching criteria and the number of controls per case |  |  |
| Variables | 7 | Clearly define all outcomes, exposures, predictors, potential confounders, and effect modifiers. Give diagnostic criteria, if applicable | 5-6 | “Preoperative assessment comprised solely of clinical questionnaires. All patients performed a preoperative self-evaluation assessment using the pain on Visual Analogue Scale (VAS), International Knee Documentation Committee (IKDC), the Knee injury and Osteoarthritis Outcome Scores (KOOS) with five subcomponents”.    “…ultrasound imagery was performed, by 5 radiologists from the same clinic, at the surgeon’s request to observe the healing of the donor site. Patients were assessed by 1 of the 5 radiologists, following a pre-established tested protocol to ensure consistent and reproducible measurements. […] The radiologists filled out a standardized form on which they noted the length, width, and thickness of the donor site defect to calculate the defect volume” |
| Data sources/ measurement | 8* | For each variable of interest, give sources of data and details of methods of assessment (measurement). Describe comparability of assessment methods if there is more than one group | 5-6 | “Preoperative assessment comprised solely of clinical questionnaires. All patients performed a preoperative self-evaluation assessment using the […] (VAS), […] (IKDC), and the […] (KOOS)”  “Patients were assessed by 1 of the 5 radiologists, following a pre-established tested protocol to ensure consistent and reproducible measurements. Additionally, one radiologist verified the measurements of another radiologist for the first two patients allocated to them. The radiologists filled out a standardized form on which they noted the length, width, and thickness of the donor site defect to calculate the defect volume.” |
| Bias | 9 | Describe any efforts to address potential sources of bias | 7 | Patients were assessed by 1 of the 5 radiologists, following the same protocol to ensure consistent and reproducible measurements. Additionally, one radiologist verified the measurements of another radiologist for the first two patients allocated to them. The radiologists filled out a standardized form on which they noted the length, width, and thickness of the donor site defect to calculate the defect volume |
| Study size | 10 | Explain how the study size was arrived at | 8 | “Post-hoc power calculations were also performed, and effect size and the sample size were deemed powerful with a 5% error.” |

Continued on next page

| Quantitative variables | 11 | Explain how quantitative variables were handled in the analyses. If applicable, describe which groupings were chosen and why |  |  |
| --- | --- | --- | --- | --- |
| Statistical methods | 12 | (*a*) Describe all statistical methods, including those used to control for confounding | 8 | “Descriptive statistics were used to summarize the findings, and Shapiro–Wilk tests were used to assess the normality of data distributions. For normally distributed continuous data, differences between the ipsi- and contralateral leg were evaluated using unpaired t-tests. Univariable linear regression analyses were performed for postoperative clinical scores, including KOOS, pain on VAS and IKDC, as well as the healing rate, using age, gender, BMI, smoking and surgical antecedents as variables.” |
|  |  | (*b*) Describe any methods used to examine subgroups and interactions | 8 | “For normally distributed continuous data, differences between the ipsi- and contralateral leg were evaluated using unpaired t-tests. Univariable linear regression analyses were performed for postoperative clinical scores, including KOOS, pain on VAS and IKDC, as well as the healing rate, using age, gender, BMI, smoking and surgical antecedents as variables.” |
|  |  | (*c*) Explain how missing data were addressed | 8 | Models were deemed sufficiently powered, considering the recommendations of Austin and Steyerberg of 2 subjects per variable. Post-hoc power calculations were also performed, and effect size and the sample size were deemed powerful with a 5% error. |
|  |  | (*d*) *Cohort study*—If applicable, explain how loss to follow-up was addressed  *Case-control study*—If applicable, explain how matching of cases and controls was addressed  *Cross-sectional study*—If applicable, describe analytical methods taking account of sampling strategy |  |  |
|  |  | (*e*) Describe any sensitivity analyses |  |  |
| Results | | | | |
| Participants | 13* | (a) Report numbers of individuals at each stage of study—eg numbers potentially eligible, examined for eligibility, confirmed eligible, included in the study, completing follow-up, and analysed | 9 | During the study period, 61 knees (61 patients; 37 males and 24 females) met the inclusion criteria. |
|  |  | (b) Give reasons for non-participation at each stage | 5 | Patients were included in the study if they underwent ACLR with a QT autograft, and had a minimum follow-up of 5 months. The authors studied a consecutive series of 61 patients (61 knees) that received ACLR using QT autograft by one surgeon between March 2019 and August 2020.The criteria for performing ACLR using QT autograft were the availability of healthy intact QT, with no antecedents of trauma, tendinopathy, or surgery. As the study aimed to investigate healing of the QT donor site, rather than the outcomes of ACLR, there were no exclusion criteria, and therefore patients with The series included 3 knees that had previous ACLR procedures or and 2 knees with multiligament injuries , which were includednot excluded, as the study aimed to investigate healing of the QT donor site at 6 months, rather than the outcomes of ACLR |
|  |  | (c) Consider use of a flow diagram |  | Figure 1 |
| Descriptive data | 14* | (a) Give characteristics of study participants (eg demographic, clinical, social) and information on exposures and potential confounders | 9 | At index surgery, the patients had a mean age of 29.8±10 years (15-54), and a BMI of 24.3±4.3 (18.6-38.8). Of the 61 knees, surgery was performed on 28 right knees (46%) and 33 left knees (54%). Meniscus repairs were required in 33 knees (54%), and 4 knees underwent revision ACLR (7%). Of the 61 patients, 21 were smokers (34%). |
|  |  | (b) Indicate number of participants with missing data for each variable of interest |  |  |
|  |  | (c) *Cohort study*—Summarise follow-up time (eg, average and total amount) |  | Table 1 |
| Outcome data | 15* | *Cohort study*—Report numbers of outcome events or summary measures over time |  | Table 5 |
|  |  | *Case-control study—*Report numbers in each exposure category, or summary measures of exposure |  |  |
|  |  | *Cross-sectional study—*Report numbers of outcome events or summary measures |  |  |
| Main results | 16 | (*a*) Give unadjusted estimates and, if applicable, confounder-adjusted estimates and their precision (eg, 95% confidence interval). Make clear which confounders were adjusted for and why they were included |  |  |
|  |  | (*b*) Report category boundaries when continuous variables were categorized |  |  |
|  |  | (*c*) If relevant, consider translating estimates of relative risk into absolute risk for a meaningful time period |  |  |

Continued on next page

| Other analyses | 17 | Report other analyses done—eg analyses of subgroups and interactions, and sensitivity analyses | 10 | “Age was significantly associated with the healing rate (β,-0.25; 95% CI, -0.48–0.02; p=0.032), while BMI was significantly associated to pain on VAS (β,0.11; 95% CI, 0.02–0.19; p=0.013) and IKDC (β, 1.12; 95% CI, (-2.13– -0.10); p=0.032).” |
| --- | --- | --- | --- | --- |
| Discussion | | | | |
| Key results | 18 | Summarise key results with reference to study objectives | 11 | “The most important finding for this study is that at a minimum follow up of 5 months, the defect size of the donor site had healed by 93±9% leaving a mean defect volume of 323.3mm^3^ according to ultrasound measurements of the donor-site” |
| Limitations | 19 | Discuss limitations of the study, taking into account sources of potential bias or imprecision. Discuss both direction and magnitude of any potential bias | 13 | “The main limitation of this study is the short-term follow up. A longer observation period is required to analyse the full QT healing process, and observe the potential residual scarring of the QT once the defect has healed in all patients, along with possible side effects. A longer follow up could allow to evaluate the evolution of the clinical scores. Additionally, while width and thickness could be assessed with no difficulty using ultrasound, in the present study, defect length was limited by the size of the ultrasound wand, resulting in shorter defect lengths than expected. Further studies are required with more adequate equipment to continue investigating the healing process of the QT. Furthermore, patients were not in the same position when harvesting the graft and when performing the ultrasound measurement at 6 months, and were assessed by 5 different radiologists post-operatively. Standardising the measurement methods would remove any potential biases for future studies. Finally, to protect the graft, no functional assessment was performed at 6 postoperative months, and therefore longer term follow up could include this assessment.” |
| Interpretation | 20 | Give a cautious overall interpretation of results considering objectives, limitations, multiplicity of analyses, results from similar studies, and other relevant evidence | 11 | QT donor site has high rates of healing, but surgeons should be aware of lower healing rates in older patients, although further studies are required to determine the impact of this finding. To the author’s knowledge, no studies have investigated the healing of the QT donor site using ultrasound imaging, and therefore this study could serve as reassurance for physiotherapists and patients who are receiving ACLR with a QT graft, as the healing process and the risk of tear is less known that other graft types |
| Generalisability | 21 | Discuss the generalisability (external validity) of the study results | 14 | “At 6 months follow up, the defect size of the QT donor site had healed by 93±9% leaving a mean defect volume of 323.3mm^3^ according to ultrasound measurements. This suggests that the QT has a high capacity for healing after graft harvesting, with 10 patients reaching full defect closure 6 months after surgery. The clinical relevance of these findings is that the QT donor site has high rates of healing, but surgeons should be aware of lower healing rates in older patients.” |
| Other information | |  | | |
| Funding | 22 | Give the source of funding and the role of the funders for the present study and, if applicable, for the original study on which the present article is based |  | Complementary file |

*Give information separately for cases and controls in case-control studies and, if applicable, for exposed and unexposed groups in cohort and cross-sectional studies.

**Note:** An Explanation and Elaboration article discusses each checklist item and gives methodological background and published examples of transparent reporting. The STROBE checklist is best used in conjunction with this article (freely available on the Web sites of PLoS Medicine at http://www.plosmedicine.org/, Annals of Internal Medicine at http://www.annals.org/, and Epidemiology at http://www.epidem.com/). Information on the STROBE Initiative is available at www.strobe-statement.org.
